# Fertility of the European Brown Hare Across Plain, Hill and Mountain Environments in the Italian Peninsula

**DOI:** 10.3390/ani14243690

**Published:** 2024-12-20

**Authors:** Pierangelo Freschi, Carlo Cosentino, Egidio Mallia, Valter Trocchi

**Affiliations:** 1 Department of Agricultural, Forestry, Food and Environmental Sciences (DAFE), University of Basilicata, 85100 Potenza, Italy; carlo.cosentino@unibas.it; 2 Ufficio Foreste e Tutela del Territorio del Dipartimento Politiche Agricole e Forestali, della Regione Basilicata, 85100 Potenza, Italy; egidiomallia2@gmail.com; 3 Federazione Italiana della Caccia (FIdC), 00198 Roma, Italy; valter.trocchi@fidc.it

**Keywords:** European brown hare, placental scars, fertility, reproduction seasonality, environmental conditions

## Abstract

This study examines the reproductive performance of the European brown hare in Italy, focusing on four hunting districts in Basilicata (A1, A2) and Emilia–Romagna (A3, A4) and using the number of endometrial scars as a fertility indicator. Fertility, measured as the number of scars during the entire reproductive period, was highest in A1 (11.50 ± 1.64) and A4 (11.43 ± 1.01), followed by A3 (9.56 ± 1.09) and A2 (6.08 ± 1.34). Significant differences were found when comparing the mountain area (A2) with A4 (*p* < 0.01) and with A1 and A3 (*p* < 0.05). Regarding the overall monthly number of scars per individual across the four study areas, the highest value (3.16 ± 0.18) was observed in May, followed by March (2.74 ± 0.18), April (2.67 ± 0.20), June (2.57 ± 0.20), February (2.53 ± 0.34), July (2.40 ± 0.22), and August (2.19 ± 0.31). A correlation analysis identified a weak yet statistically significant positive relationship (*r* = 0.532; *p* = 0.036) between the Kerner Oceanity Index (with values of 16.87, 15.00, 8.45, and 11.11 in A1, A2, A3, and A4, respectively) and the number of scars per pregnancy across the four study areas. Understanding species’ reproductive dynamics is vital for assessing habitat suitability and creating strategies to sustain hare populations and their ecosystem roles.

## Simple Summary

This study analyzes the reproductive performance of the European brown hare across different areas and environments in Italy: a hilly and a mountainous district in Basilicata and two plains districts in Emilia–Romagna. Fertility varied significantly by region, with the highest rates observed in the areas closest to the coast in Emilia–Romagna and the lowest found in the mountainous areas of Basilicata. Seasonal fertility peaked in May across all areas and showed a slight positive correlation with climate oceanity levels. These findings highlight the need for differentiated conservation strategies aimed at enhancing hare population resilience. Future research should incorporate factors like genetic diversity, juvenile survival, and environmental impacts to better understand regional reproductive differences and improve conservation efforts to ensure long-term population sustainability.

## 1. Introduction

The genus *Lepus* has a wide distribution worldwide, showcasing significant adaptability in reproductive strategies. These strategies generally consist of minimizing the reproductive cost relative to adult survival while maximizing offspring survival. Under unfavorable environmental conditions—such as with regard to climate, photoperiod, and the quality or availability of food—some hare species have evolved to adopt seasonal reproductive patterns. Notable examples include *Lepus europaeus*, *Lepus timidus*, *Lepus arcticus*, and *Lepus americanus*. Conversely, species like *Lepus corsicanus*, *Lepus granatensis*, and *Lepus capensis* maintain year-round reproduction, albeit with periods of reduced intensity [1,2,3,4,5,6,7]. Despite these reproductive adaptions, the European brown hare has experienced significant population declines across Europe, including in Italy, since the 1960s [4,8,9].

This species is characterized by a rapid turn-over of populations and high losses among the young due to natural causes—such as predation, disease, climatic adversity, and food shortages—and artificial causes, including agricultural machinery, pesticides, road traffic, and hunting, the impacts of which are highly variable in time and space. As the European brown hare is a species of interest to hunters throughout Europe, restocking activities have been carried out for a long time, either with individuals bred in captivity or with contingents caught in the wild in protected areas or imported from traditional exporting countries in Eastern Europe (primarily Hungary, Poland, and Romania) or South America (i.e., Argentina). These translocations also rearranged the original gene pool [10], with unclear consequences for reproductive performance. In Southern Italy, the presence of the European brown hare as a native species has not been proven, and its documented introduction, which began in the 1950s, took place in the historical range of the Italian hare (*L. corsicanus*).

To better understand the reproductive performances and population dynamics of the European brown hare across different habitats and regions, it is essential to analyze and compare key reproductive metrics that provide insights into reproductive health and success. Metrics such as fertility, pregnancy rate, and reproductive success are crucial for assessing this species’ reproductive efficiency. The breeding patterns and population dynamics of the European brown hare are closely tied to environmental factors, primarily photoperiodism, latitude, altitude, and food availability [6,11,12,13,14,15]. Individually, age and current and previous physical and health conditions also influence these performances [16,17]. In mid-latitude regions, the typical birth season for this species usually lasts about 8 months, beginning in January and continuing until September, with sporadic births in October and November [5,15,18,19]. In the Southern Hemisphere (namely, Argentina), where this species was introduced about 150 years ago, the breeding season extends from July to February [20,21,22,23], corresponding to the January–August period in the Northern Hemisphere. However, in Mediterranean climates, e.g., Crete [14] and Australia [24], reproduction can happen throughout the year. Conversely, European brown hares from a stock bred in Southern Italy in captivity under natural light conditions and a Mediterranean climate did not reproduce all year round [25]. This relative plasticity in the reproductive cycle of the European brown hare indicates the ability of populations to adapt to different environmental conditions, which may or may not correspond to diversity at the genetic level. Martinet [26] studied the reproductive cycles of hares bred in captivity under different lighting conditions. Under constant artificial long-day lighting (16L/8D), reproduction was maintained all year round. With increasing daylight, melatonin levels dropped, triggering sexual activity in females, with mating inducing ovulation [11,27].

Brown hares have a gestation period of approximately 41–42 days, although though superfetation births can occur 38 days apart, with frequent mating between the 34th and 40th day, peaking around days 37–38 [27]. This reproductive strategy—also observed in other genera, e.g., *Muntiacus* [28], *Mustela* [29,30], and *Panthera* [31]—enhances this species’ capacity for rapid population growth and resilience, particularly in environments where predation levels and/or environmental pressures are high. In the European brown hare, extra-uterine pregnancies have been documented [32], and embryo and fetal resorption has also been observed, which may play a role in the self-regulation of fertility [4,25].

A key factor influencing the rate of population growth is the reproductive success of females, which depends on the number of offspring they wean per litter and the frequency of litters produced annually [33]. In hare species managed for hunting purposes, it is possible to collect uterus samples to analyze uterine scars and study the fertility of individuals [34,35,36]. As reported in the literature [37,38,39], these scars—formed when the placenta separates from the uterus—remain visible in the endometrium for about 8 months [39,40,41] and indicate the number of offspring born during a reproductive period. This method of assessing fecundity has been applied in studies of rodents [42,43,44], lagomorphs [25,41,45,46,47], terrestrial carnivores [48,49,50,51,52,53,54], and seals [55].

In the present study, we evaluate the reproductive performance of the European brown hare across four distinct hunting districts in Italy, two in the north and two in the south, where the species remains relatively abundant. In particular, we focus on the analysis of fertility, understood as the number of placental scars accumulated during the entire reproductive period and their distribution in the months of the reproductive season. Comparing the reproductive performance of the European brown hare in plains, hilly, and mountainous areas can reveal insights into how varying environmental conditions impact this species’ fertility and reproductive success.

## 2. Materials and Methods

### 2.1. Study Area and Period

**Figure 1:**
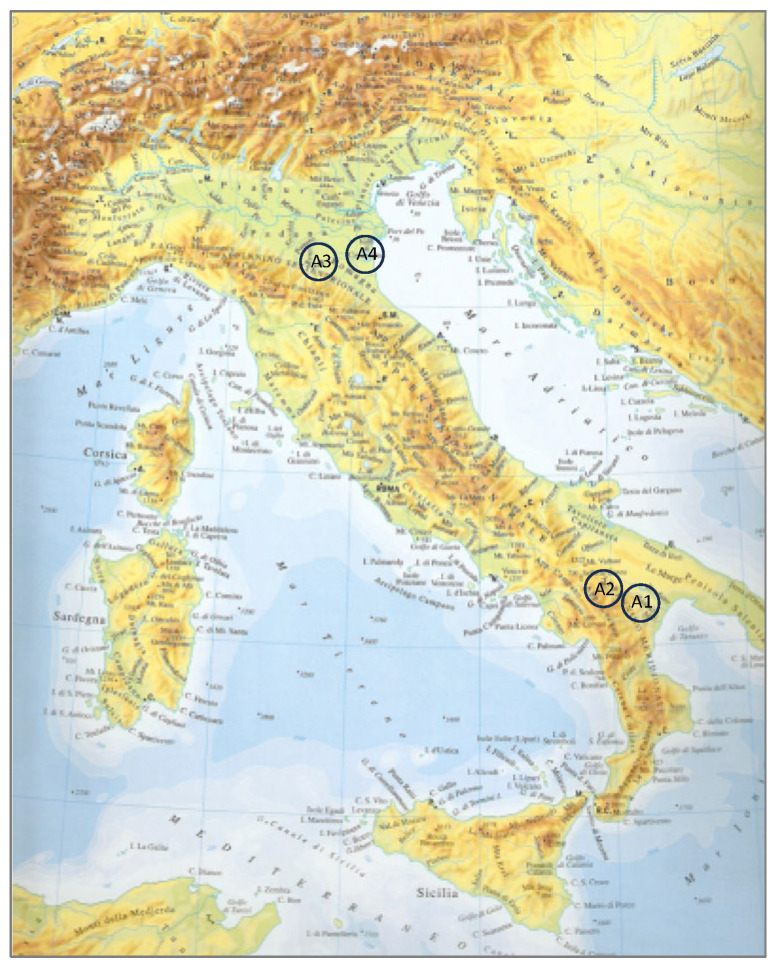
Harvesting areas: A1—hills, A2—mountains, and A3 and A4—plains.

The hares were collected in Southern Italy, in the hilly and mountainous areas of the Basilicata region (A1 and A2, respectively), and in Northern Italy, in the plains of the provinces of Bologna (A3) and Ferrara (A4) of the Emilia–Romagna region (Figure 1).

To classify the areas from a climatic perspective, the Kerner Oceanity Index (*k*_i_) [56] was calculated utilizing the mean monthly temperature in the year 2020 (Table 1). The index was determined using the following formula: *k*_1_ = 100 (T_O_ − T_A_)/E. Here, E is the annual range of monthly mean air temperatures (°C), calculated as the difference between the mean temperatures of the hottest and coldest months. This is determined by subtracting the monthly mean minimum temperature from the monthly mean maximum temperature. T_O_ and T_A_ represent the mean air temperature (°C) for October and April, respectively. Values of *k*_1_ < 10 indicate a continental climate, while values of *k*_1_ > 10 correspond to an oceanic climate [57,58,59].

### 2.2. Citizen Science Involvement

**Figure 2:**
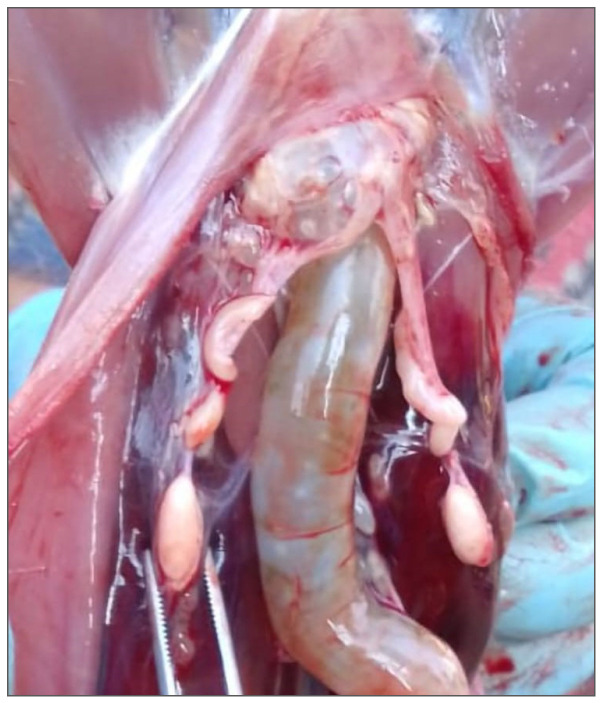
Phase consisting of the removal of the uterus from the freshly harvested hare.

Specialist hare hunters were engaged in a citizen science activity following targeted training designed to ensure accurate sampling of uteri (Figure 2).

Training consisted of theoretical and practical courses, with particular emphasis on the proper removal of uteri from the abdomino–pelvic cavity and appropriate storage methods, and was delivered by experienced staff before the hunting season commenced. Only resident hunters authorized to hunt this species in their own hunting areas participated, with 9, 6, 11, and 18 hunters from areas A1, A2, A3, and A4 participating, respectively. Moreover, in the Basilicata region, the Italian hare is present in only a few protected areas. Therefore, to prevent misclassification (which was unlikely), hunters were explicitly instructed to contact us if they had any doubts regarding species identification.

### 2.3. Sample Collection, Conservation, and Laboratory Procedures

Hares were collected in accordance with article 18 of national law No. 157/1992 in the period between the third Sunday of September and 31 October during the hunting season in 2020. The ages of hares were determined in the laboratory using Stroh’s tubercle method [60] that allowed us to discriminate young hares (yearlings) from adults. The quantities of adult fertile females utilized in this study in groups A1, A2, A3, and A4 were 15, 16, 19, and 28, respectively. Juvenile fertile females were excluded due to their low numbers and to ensure a more homogeneous sample was obtained, while adult non-reproductive females were also excluded.

**Figure 3:**
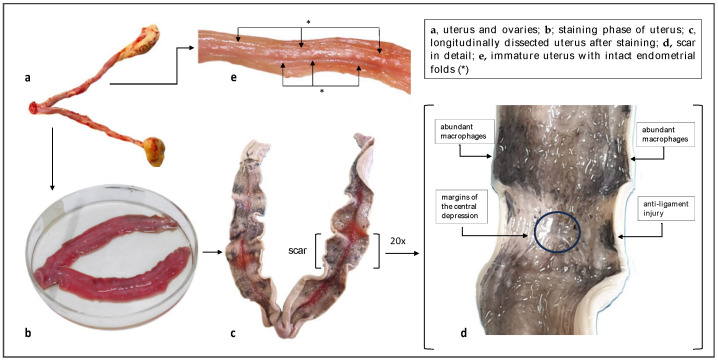
Hares’ uteri: staining phase and placental scar in detail.

After the shooting, nonpregnant uteri were removed, immersed in water in a 100 mL polypropylene container, and frozen and stored until analytical determinations were made. In the laboratory, the samples were thawed for a few minutes under running water. The ovaries, fallopian tubes, and broad ligament of the uterus were then removed. The two uterine bodies were cut open longitudinally on the opposite side of where the broad ligament was inserted to avoid damaging the scars, as placental implantation occurs in correspondence with the same ligament. The samples were stained according to the Salewski protocol [61], involving a two-phase staining process: (1) the specimen was immersed in a 20% ammonium sulfate ((NH_4_)_2_SO_4_) solution for ten minutes and then rinsed under running water; (2) after being rinsed, the sample was immersed for ten minutes in a second dye consisting of a 1% ammonium chloride (NH_4_Cl) solution and potassium ferrocyanide (K_4_[Fe(CN)_6_]3H_2_O) in a 1:1 ratio. The sample was then rinsed again. Scar analysis was performed within one hour, as the dyes and dehydration tend to rapidly alter the structure of the endometrium. In Figure 3, some phases of the laboratory procedure and anatomical details are depicted.

### 2.4. Stages of Scar Evolution

The evaluation of the morphological characteristics of the scars for their subsequent classification was performed with a binocular stereoscopic microscope. The characteristics of the scars are correlated with the time elapsed between deliveries and each hare’s death, defined as the ‘age class’ or ‘stage’ of the scar (Table 2). According to Bray [39,41], this method allows for the dating of births up to 252 days before shooting, which corresponds to six possible pregnancies (6 stages × 42 days gestation). Hence, individual fertility during the entire reproductive period is the sum of scars from different pregnancies.

**Table 2:** Criteria for estimating the age classes of scars [41].

Age Class	Central Depression		Margin	Macrophages		Anti-Ligament Injury	
	Grade	Color		Abundance	Color	Morphology	Color
1	very deep	blue-black	very clear and prominent	very abundant and grouped	blue-black	very deep and clear	blue-black
2	very deep	bronze	very clear and prominent	very abundant and grouped	bronze	very deep and clear	bronze
3	deep	brown	present and little detected	abundant and grouped	brown	deep and very clear	brown
4	shallow	brown	present and flattened	on average abundant and more dispersed	brown	shallow but visible	brown
5	slight and barely visible	beige	traces	less numerous and dispersed	beige	slight and barely visible	beige
6	flat or convex	variable or absent	absent	absent	variable or absent	absent	variable or absent

### 2.5. Statistical Analysis

The quantities of scars and litters were analyzed via the GLM procedure using a bi-factorial linear model that included the effects of the area (A1, A2, A3, and A4), the month of delivery (1–9; January to September), and their interaction. Since the interactive effect of the factors was not significant, a bifactorial model without interaction was used. Differences between means were determined using the Tukey’s HSD test. Pearson’s correlation analysis was used to assess the association between the *k*_i_ index and two parameters: ‘number of scars per pregnancy’ and ‘number of litters per breeding season’ across the four different areas. Statistical significance was set at *p* < 0.05. Before performing the correlation analysis, the normality of the variables was tested using the Shapiro–Wilk test. All analyses were carried out using SAS software, v.9.13 [62].

## 3. Results

The examination of the individual number of endometrial scars observed throughout the breeding period revealed some significant differences among the four hunting areas, except for the months of January and September (Table 3). In February, the number of scars in the districts of Basilicata was significantly lower compared to those in the Emilia–Romagna region. Specifically, the average number of scars in A1 (1.0 ± 0.68) was markedly lower than that in A3 (3.67 ± 0.56) (*p* < 0.001). Similarly, A2 hares (2.0 ± 0.68) had fewer scars compared to those from A4 (2.40 ± 0.31) (*p* < 0.05). In March, A1 hare productivity, which had been at its lowest in February, tripled (3.17 ± 0.39), reaching levels comparable to those in A3 and A4 (3.00 ± 0.43 and 2.82 ± 0.29, respectively). No significant difference was observed in April. The highest reproductive performance in A1 and A4 hares was observed in May (3.8 ± 0.43 and 3.06 ± 0.24, respectively). In June, the highest reproductive performance was observed in A4 hares (2.92 ± 0.40), which differed significantly (*p* < 0.001) from the lowest observed in A3 (1.67 ± 0.40). In the midsummer month of July, as in May, the most productive hares were observed in A1 (3.00 ± 0.48) and A4 (2.78 ± 0.37), both with a significantly higher number of scars (*p* < 0.05) compared to the A2 hares (1.67 ± 0.56). In August, significant differences (*p* < 0.05) in the number of scars were found only between the two areas of Emilia–Romagna: 2.63 ± 0.24 in A3 and 1.82 ± 0.29 in A4. In September, reproduction was observed only in A1 and A4, without significant differences.

**Figure 4:**
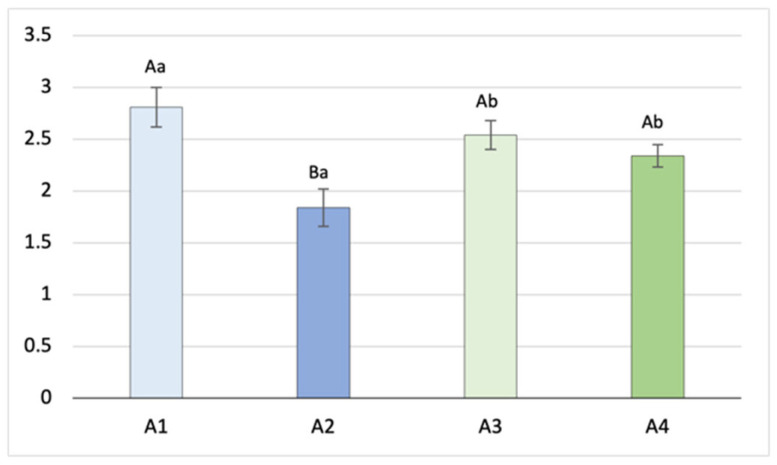
Number of scars per pregnancy (mean ± SE) on a per-area basis (a, b = *p* < 0.05; A, B = *p* < 0.001).

Figure 4 shows the average number of scars on a per-hare and -month basis in the four hunting areas during the breeding period. A significantly higher monthly frequency of scars was observed in A1 (2.81 ± 0.187) compared to that in A2 (1.84 ± 0.19; *p* < 0.001) and the areas of the Emilia–Romagna region, A3 (2.54 ± 0.14; *p* < 0.05) and A4 (2.34 ± 0.11; *p* < 0.05), in which closer performances were exhibited.

**Figure 5:**
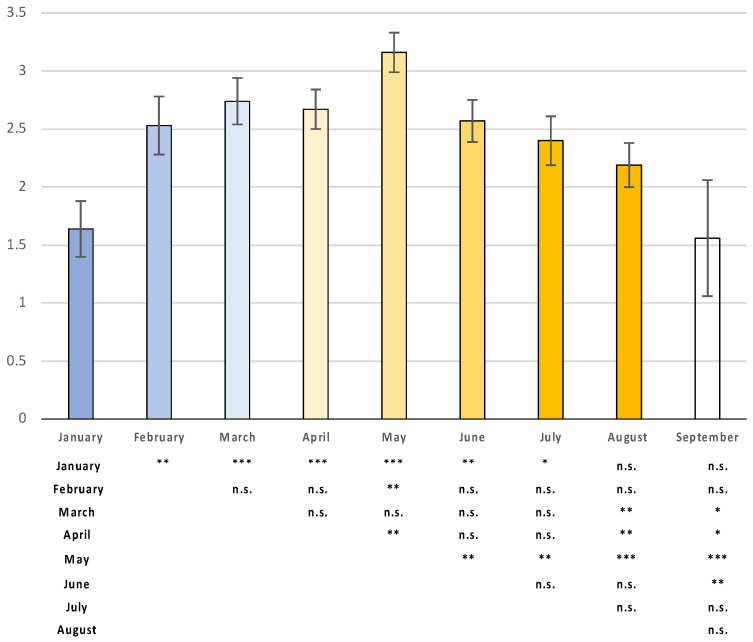
Number of scars per pregnancy on a per-month basis across all areas (mean ± SE) and significant differences (* = *p* < 0.05; ** = *p* < 0.01; *** = *p* < 0.001).

The highest number of scars per pregnancy on a per-month basis was observed in May (3.16 ± 0.18) (Figure 5), followed, in the range of > 2 < 3, in decreasing order, by March (2.74 ± 0.18), April (2.67 ± 0.20), June (2.57 ± 0.20), February (2.53 ± 0.34), July (2.40 ± 0.22), and August (2.19 ± 0.31). The lowest quantities of scars were observed in January (1.64 ± 0.44) and September (1.56 ± 0.31), although in the latter case, data were found only in A1 and A4.

**Figure 6:**
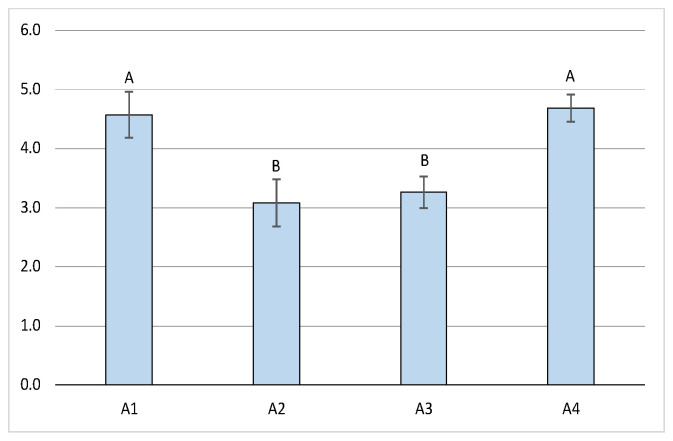
Number of litters per breeding season (mean ± SE) across the four areas (A, B = *p* < 0.01).

The mean number of litters in A1 (4.57 ± 0.39) and A4 (4.68 ± 0.23) was significantly higher (*p* < 0.01) than in A2 (3.08 ± 0.40) and A3 (3.26 ± 0.27) (Figure 6).

**Figure 7:**
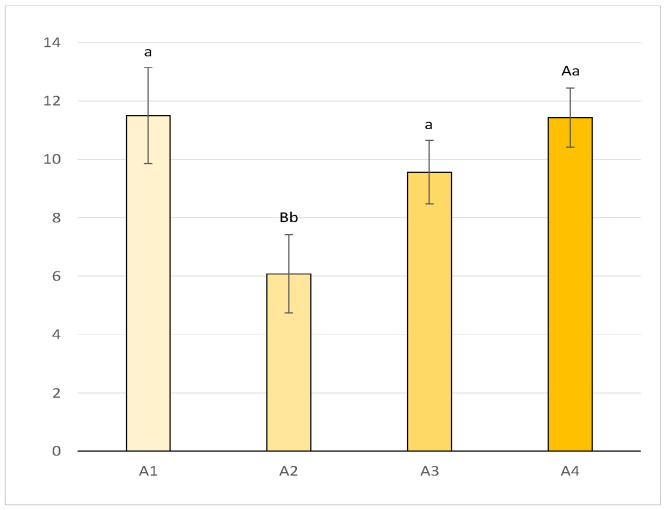
Number of scars/hare (mean ± SE) per breeding season on a per-area basis (a, b = *p* < 0.05; A, B = *p* < 0.001).

Figure 7 shows that average individual fertility during the entire reproductive period was highest in A1 (11.50 ± 1.64), followed by A4 (11.43 ± 1.01), A3 (9.56 ± 1.09), and A2 (6.08 ± 1.34). Significant differences were found when comparing the mountain area (A2) with A4 (*p* < 0.01) and with A1 and A3 (*p* < 0.05).

The correlation analysis revealed a moderately statistically significant positive correlation (Shapiro–Wilk test = 0.969, *p* = 0.119; Pearson’s *r* = 0.532, *p* = 0.036) between the *k*_i_ index (with values of 16.87, 15.00, 8.45, and 11.11 in A1, A2, A3, and A4, respectively) and the number of scars per pregnancy across the four study areas. The correlation between *k*_i_ and the number of litters was not statistically significant.

## 4. Discussion

### 4.1. Reproductive Performance and Methodology

This study examined the reproductive performance of the European brown hare across different hunting districts throughout a single reproductive period, focusing on the number and temporal distribution of endometrial scars as an indicator of fertility. Stroh’s technique allows to identify almost all the juveniles of the year if they were hunted by October, and Jensen [63] estimates that 84% of them can be recognized if collected by November. We are therefore confident that we have identified all the adult females for fertility analyses. On the other hand, only a modest percentage of the young females of the year reproduce and always with a limited number of pregnancies and placental scars [6,12,19,39]. While our study focused exclusively on adult females, as they represent the primary reproductive segment of this population, we acknowledge that this limitation may affect the generalizability of our findings to the broader population. However, even with relatively small sample sizes, our study detected significant differences, underscoring the reliability of our approach. Furthermore, we evaluated a single reproductive period, which may not fully represent long-term trends or inter-annual variability. Including additional reproductive periods or integrating other complementary metrics could provide more comprehensive insights.

The findings highlighted notable spatial and temporal variations, reflecting a complex interplay between individual traits and environmental factors influencing the reproductive biology of hares. Some authors have reported that using placental scar counts may lead to a slight overestimation of litter size at birth, likely due to cases of embryo resorption not being recognized as such [6,48,51,53,64]. Nevertheless, we can also hypothesize that there was an underestimation of embryo resorption, especially in cases further away from the moment of death of a hare. However, any such bias potentially present across all sampling sites should not have affected the comparisons between sites and periods.

### 4.2. Temporal Patterns and Seasonality

The temporal analysis of endometrial scars revealed a distinct seasonality in hare reproduction, with peak activity observed from March to May across all areas, followed by a gradual decline during the summer months, a finding consistent with previous studies [65,66]. This peak corresponded with the period of optimal environmental conditions, such as longer daylight hours, milder temperatures, and vegetation richer in nutrients, which are crucial for the survival of offspring [67,68]. The marked decline in reproductive activity towards the end of summer (August–September) reflects the natural reproductive cycle of hares, where mating and birthing are timed to align with favorable seasonal conditions for offspring [27,69,70]. This trend aligns with the well-documented biology of hares, in which the reproductive cycle is closely regulated by photoperiod and other environmental factors, particularly the availability of feeding resources [71,72,73,74].

### 4.3. Spatial Variations in Reproductive Performance

The *k*_i_ index was low (<10) in area A3, reflecting the continental climate [75] of this province of Northern Italy. Conversely, in the other three provinces, with *k*_i_ values > 10, the climate was classified as marine. The results showed significant differences in reproductive performance among the four hunting areas. Areas A1 and A4 consistently showed better reproductive outcomes, as indicated by the higher number of endometrial scars observed throughout most months. The correlation analysis revealed a moderate yet statistically significant positive correlation between the *k*_i_ index and the number of scars per pregnancy across the four study areas. Although the effect size was fair, the relationship indicated that higher levels of oceanity might be associated with a slight increase in the number of scars per pregnancy. Further investigation is needed to understand the underlying mechanisms of this correlation. In fact, the unexpectedly low reproductive performance in area A2, despite it having the highest *k*_i_ value compared to the northern sites, suggested that other factors beyond the *k_i_* index were at play. In this regard, Hackländer et al. [76] and Farkas et al. [46] evidenced that climate did not significantly influence the annual reproductive performance of female brown hares. Hackländer et al. [77] found an inverse correlation between the number of placental scars and the dry weight of the lens, which is related to the age of hare. Therefore, in older populations, fertility would be lower on average. Other factors linked to the physical conditions of adult hares may also be involved; among these are the quality of the habitat in the reproductive period and parasites. Low habitat quality driven by overgrazing and uneven livestock distribution, resulting in the overutilization of A2 sites, should be considered [78,79]. Furthermore, the reduced reproductive performance of hares at the A2 site may be linked to the high prevalence of winter cereal crops in these agricultural areas. The dominance of winter cereals could shift the nutritional bottleneck to the reproductive period, potentially impacting hare fertility. This aligns with the findings of Schmidt et al. [8] regarding brown hare populations in Denmark. Low productivity could also have been influenced by a high degree of infestation by parasites. Several authors make note of ectoparasites, mostly present in livestock grazing areas [80,81,82], while Newey et al. [16] reported that *Tricostrongylus retortaeformis* infestation results in a strong reduction in the body condition and fertility of females but does not influence their survival. This endoparasite is a commonly encountered species in the small intestine of the European brown hare in Italy (87% prevalence in Central Italy) and in other areas of Europe [17].

Such differences in reproductive traits could also be related to genetic factors. Variations in reproductive traits among populations of *L. americanus* have been observed to lead to significant genetic differences, such as differences in litter size if considering different latitudes [83]. Cretan populations of *L. europaeus* are genetically different from the remaining Greek and Central European populations, a factor that could be responsible for their different and wider reproductive cycle [14]. It is known that in Southern Italy, *L. europaeus* was introduced in recent times using hares generally imported from Eastern Europe [4]. This aspect may explain the differences in the reproductive cycles of the A1 and A2 hares compared to the Cretan populations, despite their similar Mediterranean-type climates. However, this does not seem sufficient to account for the differences between A1 and A2. Interestingly, even European brown hares bred in captivity in the same region of Southern Italy maintain autumn–winter diapause in their reproductive cycle [25]. However, further research is needed to better clarify any other factors involved.

### 4.4. Implications for Wildlife Management and Conservation

The higher reproductive output in areas A1, A3, and A4 suggests that plains and hilly areas could contribute the most to the overall stability and resilience of the species. This is consistent with the findings of Hoffmann and Smith [84] regarding the adaptability of *L. europaeus* to diverse habitats, as well as those of Reynolds and Tapper [85], who demonstrated that rural landscapes significantly influence hare population dynamics, often supporting higher densities where habitat quality is favorable. However, the lower reproductive performance in area A2 raises concerns about the long-term viability of hare populations in mountainous areas, particularly if environmental conditions continue to deteriorate [86]. Flux [66] noted that reproductive cycles in this species are sensitive to environmental constraints, suggesting that harsher climates in mountainous regions may limit reproductive success. Even the expansion of forests, which, in Italy, experienced an increase of 28% from 1985 to 2015 [87], reduces the suitability of the habitat of the European brown hare [4]. In Northern Italy, hares were found to prefer mature poplar plantations, with little underbrush, but avoided natural woodlands [88,89]. Lower hare density was documented by Panek and Kamieniarz [90] in areas with widespread forests in Polish landscapes. Only a very small proportion of forest in open agricultural areas can have a positive impact on European brown hare populations [91,92]. Habitat management could, therefore, include policies to counteract the abandonment of agricultural land in mountainous areas, improving food availability and reducing disturbance in key breeding areas, as recommended by Angerbjörn and Flux [93] in their study on leporid biology. Conservation strategies should also consider the potential impacts of climate change, which could particularly affect the survival of offspring. We highlight the importance of adaptive management as a long-term response strategy to changes in reproductive behaviors due to habitat changes, further supporting the resilience of hare populations.

## 5. Conclusions

This study reveals significant spatial and temporal variations in the reproductive performance of European brown hares, providing valuable insights for wildlife management and conservation. The findings underscore the importance of region-specific strategies that account for local ecological conditions and reproductive patterns. Conservation efforts should focus on the peak reproductive periods (March to May) and areas with high fertility potential to enhance population dynamics. This can be achieved, for example, through taking suitable measures to prevent the impact of agricultural mechanization in the spring, such as delaying the first mowing of forage and preparing spring seedbeds early in the autumn–winter period. In open areas subject to intensive cultivation, the widespread increase in shelter structures during the spring–summer period can be a very important factor in improving the survival of leverets threatened by agricultural practices and predators. In mountainous regions, targeted habitat improvements are crucial to counteract the impacts of overgrazing, agricultural land abandonment, and declining food availability. While endometrial scars are a reliable fertility indicator, evaluating the reproductive success of populations requires complementary metrics like juvenile survival rates and the density of adults in late winter. Future research should also explore the effects of genetic diversity, parasitism, predation, and habitat and climate changes on hare fertility. Understanding these complex reproductive dynamics in different environmental contexts is fundamental to define adaptive management strategies capable of ensuring the long-term conservation of hare populations and their ecosystem functions.

## Figures and Tables

**Table 1:** *k*_i_ index in the four considered provinces.

Area	T_A_	To	E	*k*_i_
1	13.10	16.40	19.60	16.84
2	10.00	13.00	20.00	15.00
3	12.70	14.50	21.30	8.45
4	15.00	18.00	27.00	11.11

**Table 3:** Number of scars per pregnancy (mean ± SE) observed in the four hunting areas ^1^.

Month of Delivery	A1	A2	A3	A4
	Mean ± SE	Mean ± SE	Mean ± SE	Mean ± SE
January	2.50 ± 0.68	-	1.83 ± 0.40	1.50 ± 0.31
February	1.00 ^B^ ± 0.97	2.00 ^b^ ± 0.68	3.67 ^Aa^ ± 0.56	2.40 ^a^ ± 0.31
March	3.17 ^a^ ± 0.39	1.75 ^b^ ± 0.48	3.00 ^a^ ± 0.43	2.82 ^a^ ± 0.29
April	3.25 ± 0.49	2.29 ± 0.37	3.00 ± 0.32	2.44 ± 0.24
May	3.80 ^a^ ± 0.43	2.50 ^b^ ± 0.34	3.43 ^a^ ± 0.37	3.06 ± 0.24
June	2.67 ± 0.56	2.33 ± 0.32	1.67 ^B^ ± 0.40	2.92 ^A^ ± 0.28
July	3.00 ^a^ ± 0.48	1.67 ^b^ ± 0.56	2.00 ± 0.37	2.78 ^a^ ± 0.32
August	2.50 ± 0.69	1.00 ± 0.97	2.63 ^a^ ± 0.24	1.82 ^b^ ± 0.29
September	2.00 ± 0.69	-	-	1.50 ± 0.69

^1^ Within one month, different letters indicate significant differences (a, b = *p* < 0.05; A, B = *p* < 0.001).

## Data Availability

Data are available on request.
